# Unravelling the Clinical Co-Morbidity and Risk Factors Associated with Agenesis of the Corpus Callosum

**DOI:** 10.3390/jcm12113623

**Published:** 2023-05-23

**Authors:** Callum J. Smith, Zoey G. Smith, Hania Rasool, Katie Cullen, Meghana Ghosh, Thomas E. Woolley, Orhan Uzun, Ne Ron Loh, David Tucker, Yasir Ahmed Syed

**Affiliations:** 1 Neuroscience and Mental Health Innovation Institute, Hadyn Ellis Building, Cardiff CF24 4HQ, UK; 2 School of Bioscience, Cardiff University, The Sir Martin Evans Building, Museum Ave., Cardiff CF10 3AX, UK; 3 School of Mathematics, Cardiff University, Cardiff CF24 4AG, UK; 4 University Hospital of Wales, Heath Park, Cardiff CF10 3AX, UK; 5 Royal United Hospitals Bath, NHS Foundation Trust, Bath BA1 3NG, UK; 6 Knowledge Directorate, Public Health Wales, Swansea SA2 8QA, UK

**Keywords:** agenesis of corpus callosum, congenital heart disorders, neurodevelopmental disorders, comorbidity, risk factors

## Abstract

Agenesis of the Corpus Callosum (ACC) can result in multiple neurological deficits including social and behavioural issues. However, the underlying aetiology, clinical co-morbidity and the contributing risk factors remain elusive, resulting in inaccurate prognosis and delayed therapy. The main objective of this study was to comprehensively describe the epidemiology and clinical co-morbidity associated with patients diagnosed with ACC. The secondary objective was to identify the factors that contribute towards increased risk for ACC. For this, we analysed 22 years (1998–2020) of clinical data across the whole of Wales, UK collected through the Congenital Anomaly Register & Information Service (CARIS) and Public Health Wales (PHW). Our results demonstrate that complete ACC (84.1%) was the prevalent subtype, in comparison to partial ACC. Further, ventriculomegaly/hydrocephalus (26.37%) and ventricular septal defect (21.92%) were identified to be the most prevalent neural malformation (NM) and congenital heart disorder (CHD) in our cohort. Although 12.7% of subjects with ACC had both an NM and CHD, we found no significant association between them (*χ*^2^ (1, *n* = 220) = 3.84, *p* = 0.33). We found socioeconomic deprivation and increased maternal age contributed towards an increased risk for ACC. To the best of our knowledge, this study for the first time defines the clinical phenotypes and the factors that contribute to ACC within the Welsh population. These findings will be of value to both patients and healthcare professionals, who may take preventative or remedial measures.

## 1. Introduction

Agenesis of the corpus callosum (ACC) is one of the most common congenital brain malformations [1], occurring when the development of the corpus callosum (CC) is disrupted due to impaired development of callosal fibres [2]. The CC is the largest interhemispheric white matter tract of the brain, containing approximately 200 million neurons [3] and develops from the 6th to 8th week of pregnancy [4], continuing until all callosal regions are formed between [5] the 18th and 22nd week of gestation [5,6]. The long development time and complexity mean that CC development is mediated by several factors [4]. The CC is vital for communication between the brain hemispheres and facilitates the management of the physical, sensory, and reasoning functions of the brain [7]. The variable size and thickness of the CC influences cognition with increased thickness correlating with higher processing speeds and intelligence in childhood, and less dense structures being associated with earlier cognitive decline in adults [8]. ACC is one outcome of disruption in CC development and can present as either a complete absence (cACC) or a partial absence (pACC) of the CC [9].

The exact incidence of ACC is not universally agreed upon because very few studies have explored the epidemiology and aetiology of ACC [10]. Previous studies illustrated that ACC incidence deviates across groups of people, with ACC thought to affect one in every 4000–5000 individuals and argued to be more prevalent in men [11]. Due to the asymptomatic effect in early life, the severity and occurrence of ACC are often misjudged [2]. The causes of ACC are complex and unclear because it can manifest as an isolated pathology, or as a component in >200 congenital syndromes [9,10]. Congenital syndromes are the biggest risk factor for ACC, with 10% of ACC cases associated with chromosomal anomalies and 20–35% of ACC cases related to specific monogenic or polygenic disorders [12,13]. ACC is rarely an isolated neurological malformation and usually presents alongside other neurodevelopmental malformations (NM). Symptoms include intellectual deficits, epileptic seizures, psychiatric problems, and socio-behavioural problems [9]. These associated NM include microcephaly and ventriculomegaly [2]. In isolated ACC cases, it is easy to conclude ACC is the cause of symptoms. However, in syndromic disorders, the opposite is true because other organs and structural malformations may influence symptoms.

Similarly, congenital heart defects (CHD) occur frequently alongside syndromic ACC [14]. The direct effects of CHD on the developing foetal brain are not well understood but may influence the formation of NM [15]. Sun et al. demonstrated those with CHD can have reduced foetal brain volume because of inefficient cerebral perfusion [16]. Therefore, CHD may play a role in the development of NM and implies a possible association between CHD and NM. There have been previous studies investigating the association between CHD and neurological abnormalities [17] and between ACC and NM and CHD, respectively; however, there are currently no studies investigating the association of NM and CHD in those with ACC.

Risk factors for developing ACC remain largely unknown, but several environmental factors have been suggested. The most common environmental factor is Foetal Alcohol Syndrome (FAS), with ACC occurring in around 7% of FAS cases [18], whilst teratogenic infections and other toxic/metabolic exposures could also contribute to ACC [13]. Research on ACC is sparse, most studies used very small sample sizes, and, in most cases, ACC was detected postnatally, in conjunction with other conditions. This potentially skews data by limiting the population from which data were collected and provides little information on neurodevelopmental factors leading to ACC [9,10]. This has contributed to limited knowledge among physicians leading to late identification, poor prenatal counselling, inaccurate prognosis and delayed targeted therapy.

To address this knowledge gap, this study aims to explore the relationship between ACC and associated co-morbidities and examine the contribution of maternal age at birth and socioeconomic background to developing ACC, using clinical data collected by the Congenital Anomaly Register & Information Service (CARIS), Public Health Wales (PHW) on all patients with ACC in Wales between 1998–2020, which to date represents one of the largest cohorts.

## 2. Methods

### 2.1. Data Source and Study Design

This study is a retrospective case series of clinical data from patients with ACC collected by CARIS, PHW over 22 years. The database receives reports from multiple sites and disciplines across Wales. The Welsh Index of Multiple Deprivation (WIMD) 2019 quintile score was also provided for each participant which is a measure of relative deprivation for small areas in Wales based on multiple factors including income, employment, health, education, housing, and physical environment. These data were provided as quintiles, with quintile 1 (Q1) being the most deprived and quintile 5 (Q5) being the most affluent [19].

### 2.2. Data Collation and Analysis

CARIS—the national registry for Congenital Anomalies and Rare Diseases Information Services for children in Wales, collects data from the earliest antenatal suspicion that malformations are present, until a child’s 18th birthday. Detection of anomalies usually starts from the dating scan (11–13 weeks), although for some lethal conditions detection may be earlier. Most brain anomalies including anomalies of the corpus callosum detected antenatally are picked up at the anomaly scan (18–20 weeks) or later in pregnancy. Data collection started on 1 January 1998.

The time of diagnosis varied from patient to patient: 115 cases were diagnosed antenatally; 10 cases were diagnosed at birth; 9 cases were diagnosed within the 1st week of life; 6 cases were diagnosed within 1 month of life; 28 cases were diagnosed within 1 year of life; 4 cases were diagnosed after 1 year of life; 3 cases were diagnosed postnatally but the time is unknown; and 27 cases were diagnosed at post-mortem. Additionally, the method of diagnosis also varied: 115 cases were diagnosed with an antenatal ultrasound, and, of these, 29 had further foetal MRI scans; 27 cases were detected or confirmed by postnatal ultrasound scan; 25 cases were detected postnatally via an MRI scan; 7 cases were detected using a CT scan postnatally, but for the remaining participants the method of diagnosis is not known.

Among the antenatally detected cases, 1.5% were identified during obstetric scans before 18 weeks, 40.0% were detected on the anomaly scan between 18 and 20 weeks, 24.6% were identified during a tertiary scan at a foetal medicine centre typically conducted between 21 and 24 weeks, 27.7% were detected during a growth scan between 24 and 40 weeks, and the remaining 6% were detected on other scans due to late booking and missed appointments.

The raw data provided contained data on epidemiology, syndromic and genetic diagnosis, the morphology of ACC, and all additional malformations for each patient, including NM and CHD. The epidemiological data included patient date of birth, birth outcome, date of death (where applicable), and gender. Gender was presented as male, female, or unknown, with unknown gender meaning the foetus was unviable or terminated before gender could be determined and the birth outcome was described as live birth, still birth, foetal loss, or termination.

Both cACC and pACC were determined based on the clinical description. When ACC was described as either ‘Complete Agenesis of Corpus Callosum’, ‘Agenesis of Corpus Callosum’ or ‘absence of Corpus Callosum’, it was assumed that these individuals had cACC. pACC was categorised based on the presence of the word ‘partial’ within the clinical description. When any condition was stated as probable, e.g., probable cACC, we assumed that the condition was present and treated it within the data. Our inclusion criterion was the presence of ACC either prenatally, at birth, or post-mortem. Due to the nature of the condition and this study, no exclusion criterion was used for the total data set.

**Figure 1:**
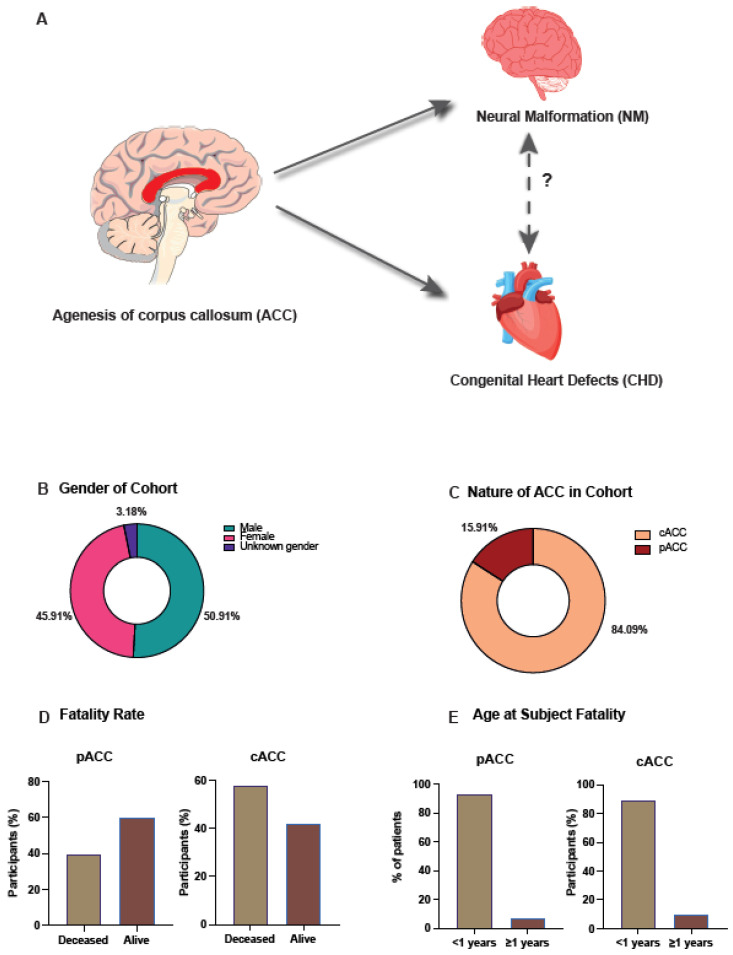
Graphical abstract and graphs depicting the demographics of study participants. (**A**) One of the aims of the study was to analyse whether there is an association between neural malformations (NM) and congenital heart defects (CHD) amongst those diagnosed with agenesis of the corpus callosum (ACC) as associations between ACC and NM and ACC and CHD have already been established. (**B**) Raw data from 220 patients with ACC provided by Public Health Wales was filtered and the data shows that 45.91% (*n* = 101) of those with ACC between 1998–2020 were female, 50.91% (*n* = 112) were male and 3.18% (*n* = 7) had unknown gender due to either foetal loss or termination of the pregnancy occurring before gender could be determined. (**C**) The ACC morphology of the cohort was determined with 84.09% (*n* = 185) showing complete ACC (cACC) and 15.91% (*n* = 35) showing partial ACC (pACC). (**D**) The fatality rate of the cohort was calculated and subsequently broken down to show the rate of fatality within each ACC morphology group. Of those with pACC 40% (*n* = 14) had passed away when the study was conducted and 60% (*n* = 21) were alive. In contrast, of those with cACC 57.84% (*n* = 107) had passed away and 42.16% (*n* = 78) were alive. (**E**) To further understand the demographics surrounding fatality of individuals with ACC the age at which the participant died was explored. 92.86% (*n* = 13) of those diagnosed with pACC died at <1 year of age. Similarly, 89.72% (*n* = 96) of the cACC cohort who had passed away died at <1 year of age.

All malformation data were provided as the condition name and with the corresponding International Classification of Disease 10 code (ICD-10), which was associated with the malformation. When a clinical description described two conditions within one data point, these were split into two data points and the missing ICD-10 codes added. NM was categorised as any neural malformation of the brain or central nervous system stated in the clinical description and the ICD-10 code. CHD was categorised as any heart defect stated in the clinical description and the ICD-10 code. When collating NM and CHD conditions, the ICD-10 code was used to enumerate the number of individuals with the condition, as there was variability in clinical descriptions and titles to name the conditions. Associations between NM and ACC and ACC and CHD were explored, in addition to investigating whether there is a statistically significant association between NM and CHD in those with ACC (Figure 1A).

CARIS provided genetic data in the form of an ICD-10 code, which was used to group genetic data and genetic descriptions for each participant. Trisomy 13–18 were grouped and enumerated under the ICD-10 code Q91, other trisomy and genetic duplication under the ICD-10 code Q92, genetic deletions under Q93, and syndromic conditions under Q87. All other genetic conditions were grouped under the title’s other conditions.

The congenital anomaly data recorded by CARIS demonstrated that the ACC population has undergone changes over the years, largely due to increased migration. Furthermore, advancements in chromosomal analysis, particularly the development of arrays, have significantly improved the ease and accuracy of testing compared to 20 years ago. Notably, the registration of abnormal karyotypes has witnessed an increase from 10.3% in 1998 to 13% in 2022, representing a proportion of all foetuses and babies with any congenital anomaly registered with CARIS.

A total of 131 patients had genetic testing completed and various methods of karyotyping were used, including 68 amniocentesis, 18 infant blood, 14 post-mortem, 10 array blood, 6 array amniocentesis, 5 specific genetic tests, 4 array post-mortem tissue and 6 other methods (including CVS & cordocentesis). Of those who underwent genetic testing 15 had trisomy 18, 4 had trisomy 13, 5 had trisomy 8, 10 involved microdeletions, 70 had a normal karyotype, 4 had unknown results, 1 failed karyotype and 5 genetic tests—(2 yielded specific results).

We compared malformation data to overall Welsh data submitted by CARIS and available from the ‘European network of population-based registries for the epidemiological surveillance of congenital anomalies (EUROCAT) between 1998–2019 [20]. This comparison was to determine if there is any difference in malformation prevalence in those with ACC in Wales compared to the wider population of congenital conditions in Wales.

### 2.3. Statistical Analysis

All data, statistical analysis and graphs were conducted and made in GraphPad Prism version 9.3.1 [21] and Microsoft Excel [22]. All statistical analysis was conducted with a 95% confidence interval and a significance level of 0.05. We used a two-tailed binomial test to determine if there is a statistically significant difference in mortality in cACC and pACC. A two-tailed binomial test was further used to determine if there is a statistically significant difference between our cohort data and EUROCAT data for NM and CHD prevalence. A chi squared (*χ*^2^) test was used to determine whether there is a significant association between NM and CHD by determining whether NM and CHD are independent, or if combinations of the categories occur more frequently than we would expect by chance, given the total number of times each category presented. A *χ*^2^ test was further used to determine whether there is a significant association between WIMD 2019 quintiles and NM and CHD diagnoses.

## 3. Results

### 3.1. Epidemiological Analysis of ACC in All Wales Cohort

Males were the most prevalent gender (50.9%), followed by females (45.9%), and 3.2% of participants had unknown or undetermined genders, (Figure 1B), this was due to either foetal loss or termination occurring before gender could be determined, as such the male to female ratio was 1:0.9. We found live birth was the most frequent birth outcome, 59.09% (*n* = 130) (Supplementary Figure S1A).

### 3.2. Complete Agenesis of the Corpus Callosum Is the Most Observed Morphology

The most frequently observed morphology was cACC (84.1%, *n* = 185) compared to pACC (15.9%, *n* = 35) (Figure 1C). To investigate whether the morphology of ACC had an effect on an individual, the number of participants who had died was investigated; 55% (*n* = 121) had passed away since birth, and of the deceased, the most prevalent morphology was cACC (88.43%, *n* = 107). Additionally, the death rates among the different morphology cohorts were explored. Of those with pACC (*n* = 35), 40% (*n* = 14) had passed away, compared to 57.84% (*n* = 107) with cACC (*n* = 185) (Figure 1D). When comparing cACC and pACC death rates to the background death rate of the cohort, we found the cACC death rate to be significantly different (*p* = 4 × 10^−6^), whereas the pACC death rate was the same as the background death rate.

To further understand the demographics of the deceased, the age at which they passed away was investigated; 92.86% (*n* = 13) of those with pACC and 89.72% of those with cACC died at <1 year of age (Figure 1E). ACC patient death was further evaluated between 1998–2020 to understand the temporal relation of death. The highest death rates were identified to occur between 2010–2013, which accounted for 22.31% (*n* = 27) of all deaths in the cohort (Supplementary Figure S1B). We found of those with diagnosed genetic conditions, trisomy 13–18 (ICD-10 code Q91) was the most observed (14.55%, *n* = 32) (Supplementary Table S1). To investigate whether there was an association between morphology and genetics, we compared the prevalence between the two morphology groups. Trisomy 13–18 (Q91) was the most common genetic condition diagnosed in those with cACC (15.68%, *n* = 29), compared to other trisomy /duplication (Q92) in those with pACC (17.14%, *n* = 6).

### 3.3. Ventriculomegaly/Hydrocephalus Was the Most Observed Neural Malformation in Those with ACC

**Figure 2:**
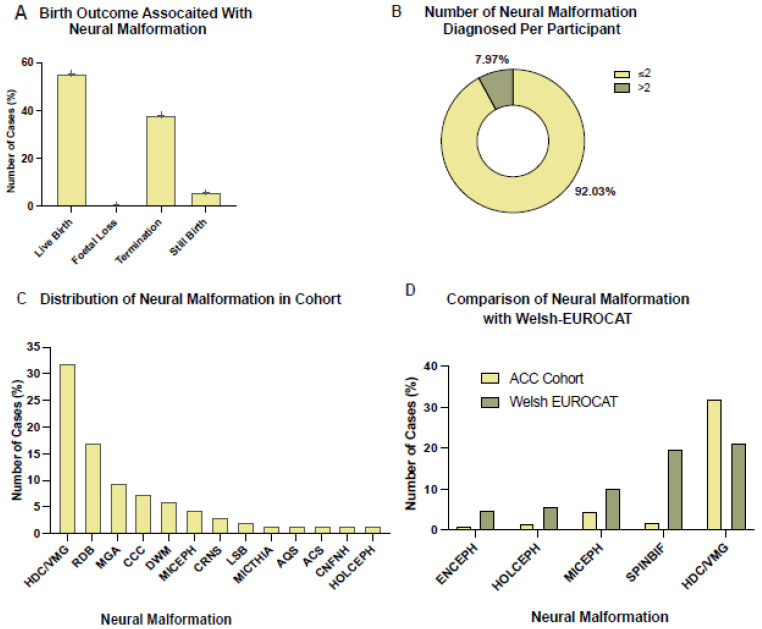
Graphs depicting the demographics of those with ACC that also had neural malformation (NM) diagnoses. (**A**) The birth outcome of those with ACC and NM (*n* = 138) was explored to investigate whether having an NM diagnosis in addition to the ACC diagnosis affected birth outcome. The data shows that live birth was the most common outcome for this cohort (55.8%, *n* = 77), followed by termination of pregnancy (37.68%, *n* = 52), then stillbirth (5.8%, *n* = 8) and lastly foetal loss (0.72%, *n* = 1). (**B**) The pie chart shows the demographics of NM diagnosis in this study’s cohort. 92.03% (*n* = 127) had two or less NM diagnoses, whilst 7.97% (*n* = 11) had more than two NM diagnoses. (**C**) The bar chart is showing which NM diagnoses were identified in this cohort and how prevalent they were amongst this cohort. Ventriculomegaly/hydrocephalus (HDC/VMG) (26.37%, *n* = 53) was the most common NM, followed by reduction deformities of the brain (ROB) (16.92%, *n* = 34), microgyria (MGA) (9.45%, *n* = 19), congenital cerebral cysts (CCC) (7.46%, *n* = 15), and Dandy–Walker malformation (DWM) (5.47%, *n* = 11). Other NM diagnoses were colpocephaly (COLCEPH) (5.47%, *n* = 11), microcephaly (MICEPH) (4.48%, *n* = 9), craniosynostosis (CRNS) (2.99%, *n* = 6), lumbar spina bifida (LSB) (1.99%, *n* = 4), micrognathia (MICTHIA) (1.49%, *n* = 3), aqueduct Stenosis (AQS) (1.49%, *n* = 3), Arnold–Chiari syndrome (ACS) (1.49%, *n* = 3), congenital malformation of face and neck, unspecified (CNFNH) (1.49%, *n* = 3) and holoprosencephaly (HOLCEPH) (1.49%, *n* = 3). (**D**) To understand whether NM occurred more or less frequently among those with ACC compared to those with ACC, the prevalence of NM among this cohort was compared to data from EUROCAT (European network of population-based registries for the epidemiological surveillance of congenital anomalies). This analysis showed that this cohort of individuals with ACC had a 3.56% higher prevalence of HDC/VMG than the EUROCAT cohort although this was not statistically significant. Spinal bifida (SPINBIF) (*p* = 4 × 10^−6^), microcephaly (MICEPH) (*p* = 0.0036), holoprosencephaly (HOLOCEPH) (*p* = 0.0055), and encephalocele (ENCEPH) (*p* = 0.0069) prevalence was significantly higher in the EUROCAT population.

ACC is rarely an isolated NM, therefore we wanted to explore associated co-morbidities; 62.73% (*n* = 138) had at least one NM diagnosed and the most common birth outcome of those with NM was live birth, 55.8% (*n* = 77) (Figure 2A). Within the NM cohort, males (52.9%, *n* = 73) were slightly more prevalent than females (43.48%, *n* = 60); cACC was the most prevalent ACC morphology (84.06%, *n* = 116); and 92.03% (*n* = 127) had ≤2 NM diagnosed, whilst 7.97% (*n* = 11) had >2 NM diagnosed (Figure 2B). We also found other trisomy/duplications; (Q92) was the most diagnosed genetic condition (12.32%, *n* = 17) of the NM cohort (Supplementary Table S1).

The effects of NM on mortality were investigated and it was found that 60.14% (*n* = 83) of those with an NM diagnosis had passed away, compared to 46.34% (*n* = 38) without an NM diagnosis. Thus, the NM cohort had a 13.8% higher rate of death; 86.75% (*n* = 72) had died at <1 year of age and cACC was the most observed ACC morphology (87.95%, *n* = 73). Moreover, amongst those that had passed away, other trisomy/duplication (ICD-10 code Q92) was most prevalent (15.66%, *n* = 13).

The most prevalent NM, excluding ACC, was found to be ventriculomegaly/hydrocephalus (HDC/VMG) (26.37%, *n* = 53) (Figure 2C) (see Supplementary Figure S2 for the incidence of NM per ACC subgroup, i.e., partial or complete ACC). We then compared the NM diagnoses to the congenital abnormalities observed in the general population of Wales using EUROCAT data. We found our cohort had a 3.56% higher prevalence of HDC/VMG than the EUROCAT population (Figure 2D), although this was not statistically significant. Spinal bifida (*p* = 4 × 10^−6^), microcephaly (*p* = 0.0036), holoprosencephaly (*p* = 0.0055), and encephalocele (*p* = 0.0069) prevalence was significantly higher in the EUROCAT population compared to our cohort (Figure 2D).

### 3.4. Ventricular Septal Defect Was the Most Observed Congenital Heart Disorder within the Cohort

**Figure 3:**
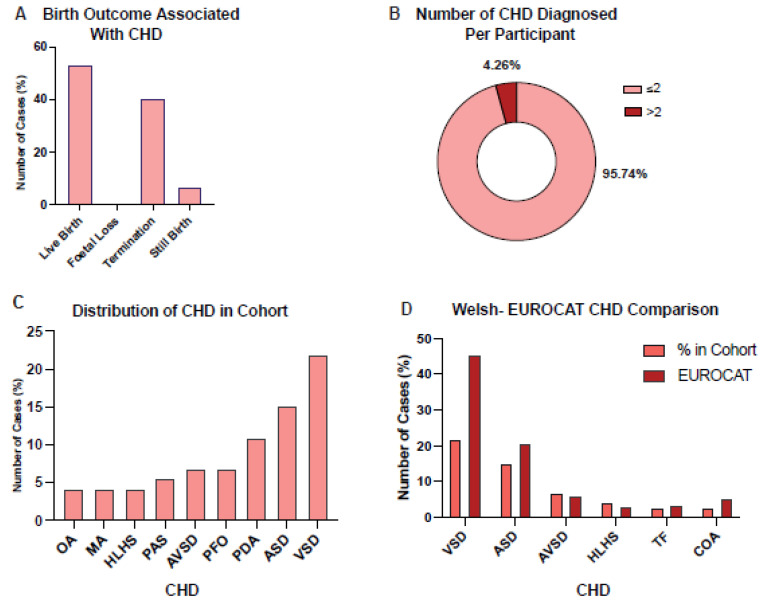
Graphs depicting the demographics of those with ACC that also had congenital heart defects (CHD) diagnoses. (**A**) The birth outcome of those with ACC and CHD (*n* = 47) was explored to investigate whether having a CHD diagnosis in addition to the ACC diagnosis affected birth outcome. The data shows that live birth was the most common outcome for this cohort (53.19%, *n* = 25), followed by termination of pregnancy (40.43%, *n* = 19), then stillbirth (6.38%, *n* = 3) and lastly foetal loss (0%, *n* = 0). (**B**) The pie chart shows the demographics of CHD diagnosis in this study’s cohort. 95.74% (*n* = 45) had less than or two NM diagnoses, whilst 4.26% (*n* = 2) had more than two CHD diagnoses. (**C**) The bar chart is showing which CHD diagnoses were made in this cohort and how prevalent they were amongst this cohort. Ventricular septal defect (VSD) was the most common CHD (21.92%, *n* = 16), followed by atrial septal defect (ASD) (15.07%, *n* = 11), and patent ductus arteriosum (PDA) (10.96%, *n* = 8). Other CHD diagnoses were, patent foramen ovale (PFO) (6.85%, *n* = 5), atrioventricular septal defect (AVSD) (6.85%, *n* = 5), pulmonary artery stenosis (PAS) (5.48%, *n* = 4), hypoplastic left heart syndrome (HLHS) (4.11%, *n* = 3), malformation of aorta (MA) (4.11%, *n* = 3) and overriding aorta (OA) (4.11%, *n* = 3). (**D**) To understand whether CHD occurred more or less frequently among those with ACC compared to those with ACC, the prevalence of CHD among this cohort was compared to data from EUROCAT (European network of population-based registries for the epidemiological surveillance of congenital anomalies). This analysis showed that the EUROCAT cohort had a significantly greater prevalence of VSD compared to our cohort (*p* = 4.7 × 10^−5^). Although, our cohort did have a significantly greater prevalence of PDA compared to EUROCAT data (*p* = 0.011). The CHDs compared were, ventricular septal defect (VSD), atrial septal defect (ASD), Atrioventricular Septal Defect (AVSD), Hypoplastic Left Heart Syndrome (HLHS), Tetralogy of Fallot (TF) and Coarctation of Aorta (COA).

We explored ACC and CHD co-morbidity and found 21.36% (*n* = 47) of the cohort had at least one CHD diagnosis and the most frequent birth outcome was live birth, 53.19% (*n* = 25) (Figure 3A). Of the CHD cohort females (51.06%, *n* = 24) were slightly more prevalent than males (44.68%, *n* = 21); cACC was the most prevalent ACC morphology (74.47%, *n* = 35); and 95.74% (*n* = 45) had ≤2 CHD diagnosed and 4.26% (*n* = 2) had >2 CHD diagnosed (Figure 3B). We also found trisomy 13–18 (Q91) to be the most observed genetic condition (31.91%, *n* = 15) (Supplementary Table S1).

The effects of CHD on mortality were investigated and it was found that 76.60% (*n* = 36) of those with a CHD diagnosis had passed away, compared to 49.13% (*n* = 85) without a CHD, thus the CHD cohort had a 27.47% higher rate of death. The demographics showed 72.2% (*n* = 26) had died at <1 year of age, cACC was the most observed ACC morphology (80.56%, *n* = 29) and, trisomy 13–18 (ICD-10 code Q91) was the most observed condition (40.67%, *n* = 15) among the deceased. We also compared the death rates of those with CHD to those who did not have CHD.

The most prevalent CHD was ventricular septal defect (VSD) (21.92%, *n* = 16) (Figure 3C) (see Supplementary Figure S2 for the incidence of CHD per ACC subgroup, i.e., partial or complete ACC). We compared our cohort to the Welsh EUROCAT database and found EUROCAT to have a significantly greater prevalence of VSD compared to our cohort (*p* = 4.7 × 10^−5^) (Figure 3D). However, our cohort had a significantly greater prevalence of PDA compared to EUROCAT data (*p* = 0.011).

### 3.5. Association between NM and CHD within ACC Cohort Was Not Significant

**Figure 4:**
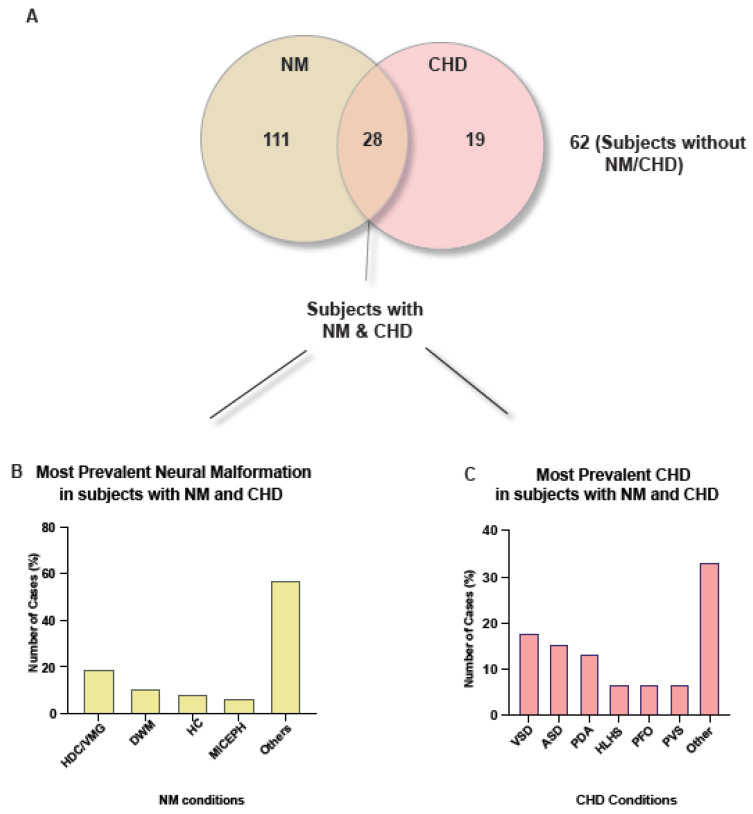
Pie chart showing the demographics of diagnoses among those with ACC and bar charts depicting the most prevalent diagnoses among those with both an NM and CHD diagnosis. (**A**) The pie chart is showing the breakdown of diagnoses across this cohort and therefore the rate of co-morbidity among the cohort. All subjects have an ACC diagnosis, with 50.45% (*n* = 111) also having at least one NM diagnosis, 8.63% (*n* = 19) having at least one CHD diagnosis, 28.18% (*n* = 62) only having an ACC diagnosis and 25.54% (*n* = 28) having both at least one NM and CHD diagnosis in addition to the ACC diagnosis. (**B**) The bar chart shows that among those with both an NM and CHD diagnosis HDC/VMG was the most frequently observed NM diagnosis (26.37%, *n* = 53). Other NM diagnoses were Dandy–Walker Malformation (DWM) (5.97%, *n* = 12), hypoplastic cerebellum (HC) (3.98%, *n* = 8), and microcephaly (MICEPH) (4.48%, *n* = 9). (**C**) The bar chart highlights that the most frequently diagnosed CHD, among those with an NM and CHD diagnosis, was ventral septal defects (VSD) (21.92%, *n* = 16). Other CHD diagnoses were atrial septal defects (ASD) (15.07%, *n* = 11), patent ductus arteriosum (PDA) (10.96%, *n* = 8), hypoplastic left heart syndrome (HLHS) (4.11%, *n* = 3), patent foramen ovale (PFO) (6.85%, *n* = 5) and pulmonary valve stenosis (PVS) (1.37%, *n* = 1).

We found that 12.7% (*n* = 28) of the cohort had both an NM and a CHD diagnosis (Figure 4A). Further, 64.29% (*n* = 18) of those with an NM and CHD diagnosis had passed away, compared to 33.33% (*n* = 21) without an NM and CHD diagnosis; thus the NM and CHD cohort had a 30.96% higher rate of death. The demographics showed 77.78% (*n* = 14) died at <1 year of age, cACC was the most observed morphology (83.33%, *n* = 15) and trisomy 13–18 (Q91) was the most observed condition (33.33%, *n* = 6) among the deceased.

Within the cohort, 57.14% had been diagnosed with <2 NM whilst 60.71% of the cohort had ≥2 CHD. Within this, HDC/VMG was the most prevalent NM (17.02%, *n* = 8) (Figure 4B) and ventral septal defects (17.78%, *n* = 8) was the most prevalent CHD (Figure 4C). As NM and CHD are observed together in 12.7% of our cohort, we wanted to determine whether there is a statistically significant association between the presence of NM and CHD; however, no statistically significant association between NM and CHD diagnosis was found, χ^2^ (1, *n* = 220) = 3.84, *p* = 0.33.

### 3.6. Risk Factors Analysis That Can Influence the Aetiology of ACC

**Figure 5:**
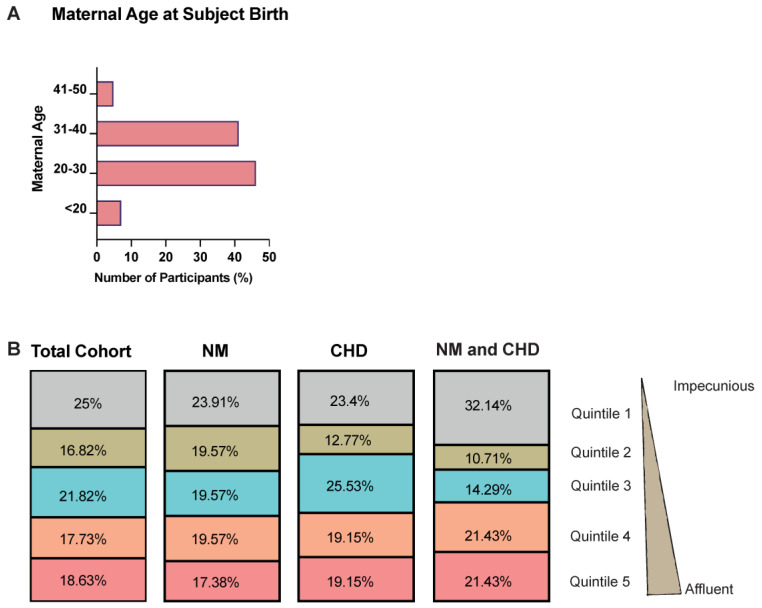
Maternal age at subject birth and socioeconomic background demographics. (**A**) One of the aims was the investigate whether maternal age is a risk factor for an individual developing ACC. As such maternal age at subject birth was explored, and the bar chart shows that the 20–30 years of age range was the most common maternal age during birth, 46.36% (*n* = 102), closely followed by the 31–40 years range, 41.36% (*n* = 91), then <20 years of age, 7.27% (*n* = 16) and finally 41–50 years of age, 5% (*n* = 11). (**B**) To further explore potential risk factors for developing ACC the WIMD 2019 quintiles of deprivation were analysed to determine if the socioeconomic background of the participants mother is associated with the development of ACC and other co-morbidities. Quintile 1, the most deprived quintile, was the most observed quintile in the total cohort (25%, *n* = 55), only NM diagnosed cohort (23.91%, *n* = 33), and both NM and CHD cohort (32.14%, *n* = 9). A chi-squared test was also conducted to determine if there is a statistically significant association between the co-morbidities diagnosed (i.e., only NM, only CHD and, NM and CHD) and the WIMD 2019 quintile. No statistically significant association (*p* = 0.45) was found between the deprivation quintiles and the co-morbidities diagnosed.

We wanted to investigate potential risk factors for ACC development. We assessed WIMD 2019 quintiles of deprivation to determine if socioeconomic background is associated with the development of ACC and other co-morbidities. Quintile 1, the most deprived quintile, was the most prevalent quintile in the total cohort (25%, *n* = 55), only the NM diagnosed cohort (23.91%, *n* = 33), and both the NM and CHD cohort (32.14%, *n* = 9) (Figure 5B); however, no statistically significant association (*p* = 0.45) between the deprivation quintiles and the co-morbidities diagnosed was found.

We also explored the ages of the mothers at the time of birth. Twenty to thirty years of age was the most common maternal age 46.36% (*n* = 102) (Figure 5A). To further understand the socioeconomical background of the cohort, we compared the ages of the mothers at the time of birth to the WIMD 2019 quintiles. We found quintile 1 to be the most observed quintile in the <20 years (50%, *n* = 8) and 20–30 years (27.45%, *n* = 28) age group. Quintile 5 was the most observed quintile in the 31–40 years (25.27%, *n* = 23), and quintile 3 (27.27%, *n* = 3) and 5 (27.27%, *n* = 3) were the most observed in the 41–50 years age group (Supplementary Table S2).

## 4. Discussion

This retrospective study of clinical data collected by CARIS over 22 years found a series of differences across the ACC cohort, with ACC being more prevalent in males and cACC the most observed morphology. Further analysis highlighted maternal age at birth and socioeconomic background may contribute towards risk factors for developing ACC. Moreover, a two-tailed binomial test showed those with cACC are more likely to die compared to the background death rate. Whilst a *χ*^2^ analysis showed no statistically significant association between NM and CHD in those with ACC, the most common NM and CHD were identified. These findings provide starting points for further research to see whether these results are seen outside of Wales which could help guide medical practitioners in diagnosis and ensure individuals with ACC get access to the correct targeted therapies in a timely manner.

We found a minor difference in the ratio of males to females diagnosed with ACC (1:0.9), whereas other studies have shown a significantly greater difference. For example, Taylor and David had 64.2% male participants and 35.7% female and therefore a difference of 28.5% [23]. This difference may be explained by differences in the number of participants, as Taylor and David had under 100 patients whilst our dataset recorded 220 patients. However, our findings were consistent with another study conducted by Romaniello et al., which found a Male:Female ratio of 1:0.8 [10]. These findings are further supported by a large-scale epidemiological study conducted in the United States, which that found 52% of their cohort were male [14]. Accordingly, the findings from this dataset imply males are potentially more likely to develop ACC but the occurrence of ACC in males and females is comparatively close. Several studies identified cACC as the most prevalent morphology [2,10,24,25] verifying the findings from this study. However, Bedeschi et al. found pACC to be more prevalent (52.38%) in their cohort of 63 participants [12]. Identifying which morphology is more prevalent can be difficult because cACC symptoms are more severe and therefore diagnosis is more likely compared to pACC diagnoses where symptoms are not as apparent [24] and the asymptomatic presentation may lead to missed diagnoses [26]. Moreover, our classification of ACC morphology assumed that unless ‘partial’ was mentioned in the clinical description, the diagnosis was cACC. This means we may have overestimated the prevalence of cACC due to vague clinical descriptions, and this is also a limitation of all research being conducted into ACC, as there is no agreed method across researchers on how cACC or pACC is described.

Other studies have investigated patients’ survival and death rates but have not investigated it as extensively as this study. Fatality of those with cACC is significantly higher than the background rate of death which implies a link between fatality and ACC morphology. However, as there were more cACC patients, it can be suggested this finding may be the result of this bias in the data. Consequently, the validity and reliability of this finding is in question.

The mothers’ age at childbirth was analysed to investigate if this is a risk factor for developing ACC. The data showed most ACC patients were born to mothers who were 20–30 years old. Similarly, other studies found average maternal ages of 28.48 and 20.5 [27,28]. This indicates our findings are replicable, but no conclusion can be made further than this as age was not standardised in this study. The most prevailing outcome of pregnancy was live birth followed by termination which is corroborated by other studies, such as Ozyüncü et al. which found the same results [27]. This shows ACC individuals can have live births and is helpful in providing information to parents whose babies have been diagnosed with ACC as it may help them decide whether they wish to continue with the pregnancy or not. Further analysis would be useful for parents in assessing and presenting exclusively the number of live birth patients that are still alive and how long they live after birth. Larger patient records would strengthen and validate this next step in research.

The prevalence of NM co-morbidities among the data set was 63% and the most prevalent malformation was ventriculomegaly (23%). The most prevalent malformations seem to be linked to cortical (ventricle-related mostly) or cerebellar neurodevelopmental disorders which is support by Hetts et al. who found malformations of cortical development were the most frequent co-morbidity and identified in 23% of participants [29]. Profiling the most prevalent of these was done to provide a useful information for clinicians. Knowing the above will help direct imaging efforts, so cases will be diagnosed earlier, and targeted therapy can begin faster. Co-morbidity of ACC and CHD was explored, and the most prevalent CHD was identified as Ventricular septal defect (VSD). This result is reliable because other studies have found similar results; for example, Wu, He and Shao found that VSD was the most common CHD cohort between 1990–2017 [30].

These results will provide further information to direct future research and give clinicians more information to discuss with parents of those with ACC, which will help parents decide if they want to continue with the pregnancy or not. Moreover, it will help clinicians to guide diagnostic testing and may assist in indicating which co-morbidities should be investigated first as well as helping to make decisions with regard to treatment interventions. It must be noted however, that whilst these results show the comorbidity between different genetic conditions and the presence of ACC in individuals, the results do not show a causative relationship between the presence of ACC in an individual and the development of a genetic condition.

Our study was the first to demonstrate there is no statistically significant association between NM and CHD in an ACC cohort. However, our finding may be limited by low statistical power because the cohort size was only 28 participants, and, therefore, the rejection of the hypothesis needs to be interpreted with caution. As ACC diagnosis is rare, a suggestion for future studies is for data to be collected on a larger scale than in Wales alone. This would allow for greater statistical power and will either support or reject the findings of this study and provide reliable information for parents. Further, this study is the first study to investigate a correlation between the socioeconomic background of patients and ACC. Previously ACC occurrence in different countries has been compared [2], but considering the level of deprivation and the number of cases reported per level of deprivation is a new finding. Quintile 1, the most deprived quintile, was the most observed quintile in the total cohort, NM-diagnosed cohort, and NM and CHD-diagnosed cohort. However, no statistically significant association between the deprivation quintiles and diagnoses were found. It would be useful for future studies to look at larger and more diverse samples to either confirm or challenge this finding. A further limitation is that the cities, towns, and areas in Wales are unknown. Knowing this socioeconomic information would allow for in-depth exploration for links between the location, supply of water, educational achievements, salary, crime, etc. and whether these environmental factors could be risk factors for developing ACC and related co-morbidities.

This analysis only looked at patients in Wales and exploring a cohort of all UK patients would enable findings to be generalised to the whole population. Investigations exploring the socioeconomic background of patients in greater depth will hopefully shed light on the impact of environmental factors on ACC causation and help prevent the development of ACC. Future analysis of ACC cohorts should include the ethnicity of patients, to explore whether ethnicity is a risk factor. To expand upon current knowledge, investigations into the genetic association between NM and CHD, using a similar exosome splicing protocol to Homsy et al. on a national cohort of participants diagnosed with ACC would be beneficial [31]. If there is a genetic association between NM and CHD, as suggested by the findings of Homsy et al. [31], this will give us a greater understanding of the mechanistic development of these co-morbidities and how they interact.

Furthermore, the high prevalence of cortical malformations over brain midline associated anomalies could suggest the mechanisms involved in neuronal migration, organisation and axonal guidance play a stronger role in ACC than those involved in midline patterning. Hence, carefully looking at the neural malformations themselves, and then looking at co-morbidities not directly related to the CNS, such as congenital heart abnormalities and other developmental disorders, and integrating this information to form links between the three is a highly desirable future outcome. Next-generation sequence processing approaches would then be required to better investigate the molecular processes causing ACC.

## 5. Conclusions

22 years’ worth of data were analysed and new insights into ACC not previously addressed have been identified. This study has helped further characterise the neural aspects of ACC by focusing on structural neural co-morbidities and the split between partial and complete ACC, in addition to characterising congenital heart defects. Findings such as identifying ventriculomegaly and VSD as the most prevalent NM and CHD co-morbidities and understanding that the most prevalent NM often pertain to the cortex and cerebellum will be useful in prenatal counselling, diagnosis, and early treatment.

A better understanding of the causes of ACC, and its links to NM and CHDs, may reduce the unpredictability of this condition, providing the potential for prospective parents to make a more informed decision regarding proceeding or terminating a pregnancy, and providing clinicians with a better basis on which to determine treatments and help to streamline diagnostic testing.

## Data Availability

The data that supports the findings are available upon request from corresponding author.

## Supplementary Materials

The following supporting information can be downloaded at: https://www.mdpi.com/article/10.3390/jcm12113623/s1, Figure S1: Birth Outcome, Incidence of ACC Diagnosis and Death of ACC Patients; Figure S2: Incidence of Neural Malformations (NM) and Congenital Heart Disorders (CHD) in those with ACC; Table S1: Incidence of Genetic Conditions in those with ACC; Table S2: Distribution of Maternal Age across Deprivation Quintiles.
