# Supplementary material for: Unravelling the Clinical Co-Morbidity and Risk Factors Associated with Agenesis of the Corpus Callosum
Source: J Clin Med. 2023 May 23;12(11):3623. doi: 10.3390/jcm12113623 (PMC10253475; doi:10.3390/jcm12113623)
Supplement: Supplementary file 1 [file jcm-12-03623-s001.zip › jcm-2368494-supplementary.pdf]

Supplementary Information

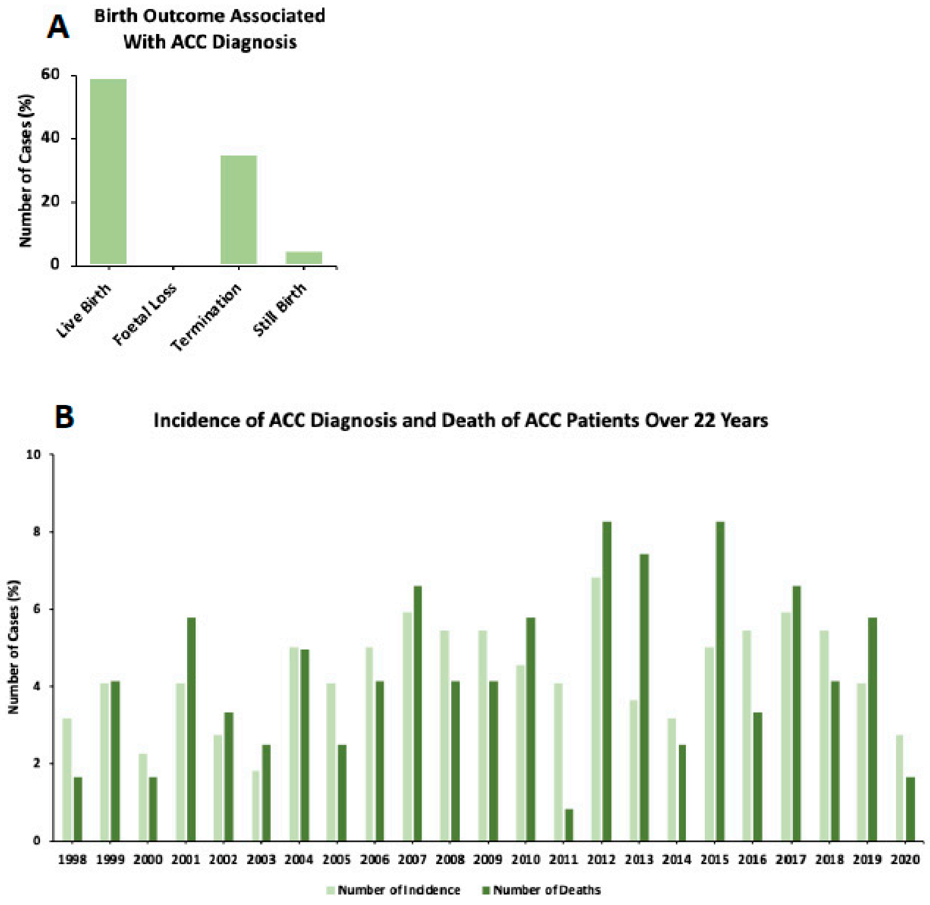

Figure S1. Birth Outcome, Incidence of ACC Diagnosis and Death of ACC Patients. **A.** The bar chart is showing the different birth outcomes for those who had been diagnosed with ACC (n=220). Live birth (59%, n=130) was the most common, followed by termination (35%, n=77), still birth (5%, n=11) and finally foetal loss (<1%, n=2). **B.** The bar chart is showing the incidence of ACC diagnosis across the 22-year period over which the data was collected by Public Health Wales, in addition to the incidence of death of those who had been diagnosed with ACC over the 22-year period from 1998-2020.

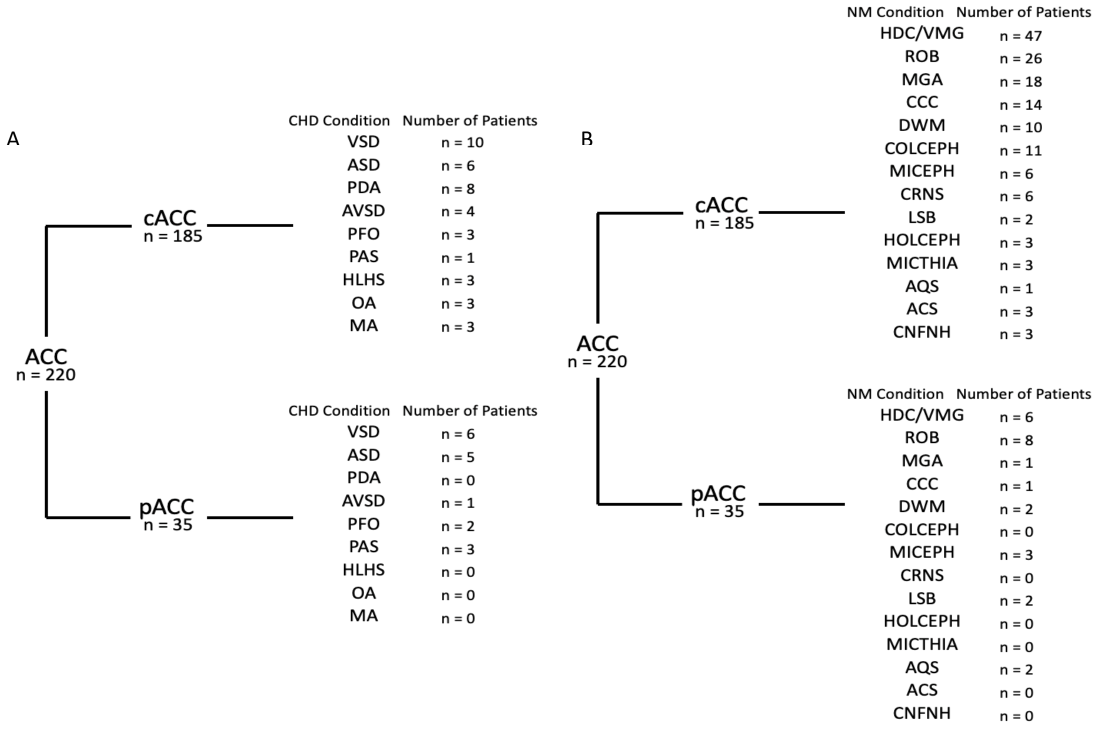

Figure S2. Incidence of Neural Malformations (NM) and Congenital Heart Disorders (CHD) in those with ACC. The flow diagrams show the incidence of NM and CHD for those with partial ACC (pACC) or complete ACC (cACC). The number of patients is indicated by n =. A). The incidence of CHD in patients with pACC and cACC. Abbreviations: ventricular septal defect (VSD), atrial septal defect (ASD), patent ductus arteriosum (PDA), patent foramen ovale (PFO), atrioventricular septal defect (AVSD), pulmonary artery stenosis (PAS), hypoplastic left heart syndrome (HLHS), malformation of aorta (MA), overriding aorta (OA). B). The incidence of NM in patients with pACC and cACC. Abbreviations: ventriculomegaly/hydrocephalus (HDC/VMG) (26.37%, n=53), reduction deformities of the brain (ROB), microgyria (MGA), congenital cerebral cysts (CCC), Dandy Walker malformation (DWM), colpocephaly (COLCEPH), microcephaly (MICEPH), craniosynostosis (CRNS), lumbar Spina bifida (LSB), micrognathia (MICTHIA), aqueduct Stenosis (AQS), Arnold-Chiari syndrome (ACS), congenital malformation of face and neck, unspecified (CNFNH) and holoprosencephaly (HOLCEPH).

Table S1. Incidence of Genetic Conditions in those with ACC. The table shows the incidence of genetic conditions in those with ACC, which have been grouped under the overarching ICD-10 codes. The incidence of genetic conditions in show in the total ACC cohort (n=220), the Neural Malformations (NM) cohort (n=138), and the Congenital Heart Defects (CHD) cohort (n=47). Excluding Z13 which is Special screening examination, Q91 is the most common genetic condition in the total ACC cohort and CHD cohort, whilst Q92 is the most common genetic condition in the NM cohort. Z13: Special screening examination for other diseases and disorders; Q99: Other chromosome abnormalities; Q98: Other sex chromosome abnormalities, male phenotype, not elsewhere classified; Q97: Other sex chromosome abnormalities, female phenotype, not elsewhere classified; Q96: Turner syndrome; Q95: Balanced rearrangements and structural markers, not elsewhere classified; Q93: Monosomies and deletions from the autosomes, not elsewhere classified; Q92: Other trisomies and partial trisomies of the autosomes, not elsewhere classified; Q91: Trisomy 18 & 13; Q89: Other congenital malformations, not elsewhere classified; Q87: Other specified congenital malformation syndromes affecting multiple systems; Q86: Congenital malformation syndromes due to known exogenous causes, not elsewhere classified; Q78: Other osteochondrodysplasias; Q44: Congenital malformations of gallbladder, bile ducts and liver; Q04: Other congenital malformations of brain; G31: Other degenerative diseases of nervous system, not elsewhere classified; C44: Other malignant neoplasms of skin.

Table S1

ICD-10 Code	Total Cohort (Count)	Total Cohort (Percentage)	NM Cohort (Count)	NM Cohort (Percentage)	CHD Cohort (Count)	CHD Cohort (Percentage)
**Z13**	50	22.73	29	21.01	3	6.38
**Q99**	3	1.36	3	2.17	1	2.13
**Q98**	1	0.45	1	0.72	0	0.00
**Q97**	2	0.91	2	1.45	0	0.00
**Q96**	2	0.91	1	0.72	1	2.13
**Q95**	3	1.36	3	2.17	0	0.00
**Q93**	17	7.73	11	7.97	4	8.51
**Q92**	24	10.91	17	12.32	6	12.77
**Q91**	32	14.55	14	10.14	15	31.91
**Q89**	2	0.91	1	0.72	0	0.00
**Q87**	8	3.64	5	3.62	4	8.51
**Q86**	3	1.36	3	2.17	2	4.26
**Q78**	1	0.45	1	0.72	0	0.00
**Q44**	1	0.45	1	0.72	1	2.13
**Q04**	1	0.45	1	0.72	0	0.00
**G31**	1	0.45	1	0.72	0	0.00
**C44**	1	0.45	1	0.72	0	0.00

Table S2. Distribution of Maternal Age across Deprivation Quintiles. The table shows the distribution of maternal age across the different deprivation quintiles. Maternal age of 20-30 years was most common in Quintile 1, 2 and 4. Maternal age 20-30 years and 31-40 years were jointly most common in Quintile 3. Maternal age of 31-40 years was the most common in Quintile 5. Of those mother <20 years of age, the most quintile for these mothers was Quintile 1, compared to Quintiles 3 and 5 for mothers aged 41-50 years old.

Table S2

	Quintile (Q) of Deprivation Based on Welsh Index of Multiple Deprivation					
**Maternal Age Range**	**Q1**	**Q2**	**Q3**	**Q4**	**Q5**	**TOTAL**
**<20**	8	2	3	1	2	16
**20-30**	28	20	21	20	13	102
**31-40**	17	14	21	16	23	91
**41-50**	2	1	3	2	3	11
**TOTAL**	55	37	48	39	41	220
